# The effect of different milk pretreatment methods on microbiome community development during Herrgårds cheese production and ripening

**DOI:** 10.1371/journal.pone.0350187

**Published:** 2026-07-14

**Authors:** Juan Antonio Rodríguez, Luisa Santos-Bay, Apurva Narechania, Christian Carøe, Kimmo Sirén, Sarah S. T. Mak, Ida Broman Nielsen, Max Ramsøe, Thomas S. Pontén, Søren Lillevang, Lene Tranberg Andersen, M. Thomas P. Gilbert

**Affiliations:** 1 Center for Evolutionary Hologenomics, The Globe Institute, University of Copenhagen, Copenhagen, Denmark; 2 Institute for Comparative Genomics, American Museum of Natural History, New York, New York, United States of America; 3 Novonesis A/S, Hvidovre, Denmark; 4 Centre of Excellence for Omics-Driven Computational Biodiscovery, Faculty of Applied Sciences, AIMST University, Bedong, Kedah, Malaysia; 5 Arla A/S, Viby, Denmark; 6 University Museum, NTNU, Trondheim, Norway; Yantai Institute of Technology, CHINA

## Abstract

One of the biggest challenges for dairy producers is the substantial variability in final product properties caused by changes in the production environment. In cheese production, this variation is influenced by several factors, particularly the milk base and its pretreatment, which shape the microbiome throughout the process and ultimately affect the cheese’s organoleptic characteristics. To examine the impact of three different pretreatments for pasteurised milk— microfiltration, protein fortification, and pasteurisation only (control)— on microbiome dynamics, we generated metagenome sequencing data from 14 cheese production steps across these three production trials at a Danish dairy factory. We constructed three metagenomic co-assemblies, identifying nine high-quality metagenome-assembled genomes. Our analysis revealed that a specific strain of *Lactococcus lactis* dominates the process, while other minor bacterial species persist at very low abundances (<1%), contributing non-negligibly to product properties. Notably, we detected DNA from *Clostridium tyrobutyricum*, a known bacterium whose heat-resistant spores may cause dairy spoilage, in pasteurised only and protein-fortified milk trials but was nearly absent in microfiltered milk. To enhance our analyses, we implemented KHILL, a novel *k*-mer based method, which facilitates metagenomic co-assembly and enables early detection of unwanted microorganisms. Our findings provide industrial dairy producers with a comprehensive view of microbial dynamics during cheese production, offering insights to improve process consistency and product quality.

## 1. Introduction

Cheese has been produced through coagulation of the animal milk protein casein for at least 7,500 years [1]. Over the last century its production has largely transitioned from an artisanal and manual cheesemaking process to a more industrialised one, linked to factories that are able to produce the volumes needed to fulfil the demands of its consumer base. However, a major challenge that industrial cheese factories often face is the lack of product homogeneity when the production environment changes — for example, when cheese manufacturing is moved to a different facility or location [2]. This is critical to industrial production, as even small changes in the process may have major consequences on the resulting organoleptic and rheological properties of cheese. Almost certainly, differences that arise in the environmental microbial communities during the manufacturing process play an important role in this variance [3,4]. In cheese production, starter cultures that contain lactic fermenting microbes are added to the milk in a controlled and standardised way. However, other sources of microorganisms, like those naturally present in the milk or even those in the facility’s immediate indoors environment and the vicinity, may well enter the process and contribute to shaping the quality and properties of the final cheese. Factors that can alter the microbial communities include: the dairy where the cheese is made, the starter culture used, differences in equipment in the facilities, and even the sources of, and pretreatment of, the milk.

Pretreatment of milk is an increasingly common procedure in the dairy industry [5]. However, the effect of pretreatment technologies, such as microfiltration and protein fortification is underexplored with regards to their effects on the cheese microbiome. In light of this, we initiated a study to profile how the microbiome community develops throughout a pilot plant trial mimicking an industrial continental cheesemaking process, as conditioned by the use of differentially treated starting milk bases, specifically microfiltration and protein fortification. Additionally, as the physical location in the cheese from which samples are taken has been shown to bias downstream microbiome analyses [6], this was considered as a factor in our analyses.

**Fig 1:**
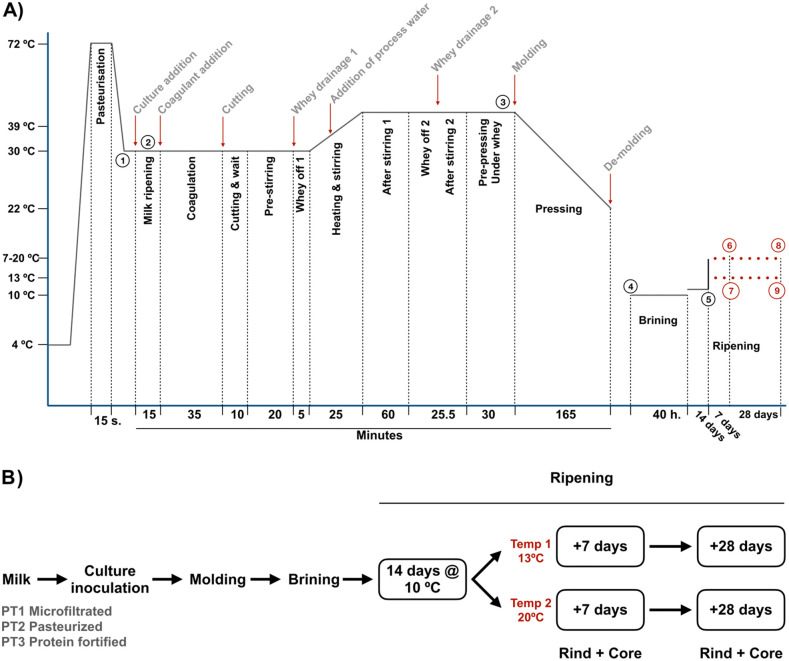
Representative scheme of the sampling process and experimental setup for the production trials. A) Diagram showing temperature and time throughout the cheesemaking process, indicating the sampling points where our samples were taken. B) Schematic diagram depicting the experimental setup for the trials. Sampling points n = 14.

Thanks to recent developments in culture-independent microbial community profiling [7,8], when adequate samples are available for analysis, it is possible to reconstruct both the species within microbial communities, and the genes present in such microbes, at a level that was previously unachievable. A decade ago, studies relied mainly on cultivation or 16S rRNA gene amplicon [9] sequencing to identify taxa, but shotgun metagenomic sequencing and computational approaches have significantly enhanced our understanding of genome-resolved environmental metagenomics [10]. In the food industry, these techniques have revolutionised our knowledge of microbial dynamics and their role in food production [11]. Combined with the increasing accessibility of multi-omics data, they provide detailed insights into microbiome dynamics. Despite previous studies describing changes in the cheese microbiome during production [2,6,12], a systematic evaluation of how milk pretreatment affects microbiome dynamics remains lacking. Thus, in light of the need to better understand the factors that help shape variation in the industrial cheesemaking microbiome, we applied this approach to the production process of Herrgårds cheese at a single pilot plant facility in Skejby (Århus, Denmark), in order to explore how variation of key production parameters shape the relationship between the microbiome and the final cheese product. Herrgårds is a traditional Swedish hard cheese made from cow’s milk, with a pale to golden-yellow interior and small, evenly distributed holes or “eyes” throughout the cheese. It has been described as being mild, nutty and creamy to taste [13]. Specifically, we generated and compared the microbiome profiles for three pilot plant production trials (PT1, PT2, PT3) of the cheesemaking process, taking samples at the same processing time points for all three PTs. Each PT was sampled at equivalent processing time points, including rind and core during ripening, incorporating two ripening temperatures and two ripening durations. In each of the three PTs the pasteurised milk underwent a different treatment, namely: microfiltration (PT1), pasteurisation only (PT2) and protein fortification (PT3). (Fig 1, see Materials and Methods section).

The main objective of the present study was to determine how different milk pretreatments shape the microbial community dynamics of Herrgårds cheese during production and ripening. Specifically, the study aimed to characterise microbiome dynamics across 14 processing stages using shotgun metagenomics. A further objective was to evaluate whether the presence of potentially detrimental microorganisms known to contribute to milk spoilage, particularly *Clostridium tyrobutyricum*, differed among the different pretreatments. Lastly, we aimed to validate the usefulness of KHILL [14], a novel computational approach based on *k*-mer counting over raw sequencing reads, as a rapid tool to detect microbial shifts, guide co-assembly procedures, and support early spoilage monitoring in industrial cheesemaking processes.

Our study uses a relatively simple cheese (compositionally speaking) in a very controlled industrial environment. However, our findings may help the dairy industry ensure robust production of premium quality cheeses when different milk pretreatments are applied. This in turn will increase not only the cost-efficiency of cheese production in general, but also the overall cheese quality and in the end also benefiting the consumer.

## 2. Results and discussion

### 2.1. Initial shotgun metagenomics data preprocessing

We generated shotgun metagenomic sequencing data from 14 different subsamples (Fig 1,Table 1) taken during each of three pilot production trials of Herrgårds cheese, yielding 42 samples in total (see Materials and Methods section for sampling details). After DNA extraction, shotgun microbiomes were sequenced using 2 × 100 bp paired end Illumina sequencing, with an average output of 52.5 M (s.d.: 15.2 M) pairs of reads generated for each of the 42 libraries. Reads were trimmed to remove sequencing adapters, leaving an average of 99.43% of the raw reads (S1 Table in S1 File). Afterwards, endogenous host DNA (cattle; *Bos taurus*) was removed by mapping against the cow reference genome (ARS-UCD1.3, *bosTau9*). Up to 99% of the reads were removed in early raw milk stages, while removing <1% during ripening (S1 Table in S1 File).

**Table 1:** Sampling description.

Description	Sampling point ID	Sample type	Cheese sampling spot	Num in Fig 1A	Sample number	Date collected		
						PT1	PT2	PT3
**Pre-inoculation, in vat**	Pre-inoculation	MILK	N/A	1	1	20/8/19	20/8/19	20/8/19
**Post-inoculation, in vat**	Post-inoculation	MILK	N/A	2	2	20/8/19	20/8/19	20/8/19
**Before moulding**	Before moulding	GRAINS	N/A	3	3	20/8/19	20/8/19	20/8/19
**Before brining**	Before brining	CHEESE	N/A	4	4	20/8/19	20/8/19	20/8/19
**Temp 0 – After 14 days at 10ºC**	T0_10C_Rind	CHEESE	Rind	5	5	5/9/19	5/9/19	5/9/19
**Temp 0 – After 14 days at 10ºC**	T0_10C_Core	CHEESE	Core		6	5/9/19	5/9/19	5/9/19
**Temp 1 – After 7 MORE days at 13ºC**	T1_7d_13C_Rind	CHEESE	Rind	6	7	12/9/19	12/9/19	12/9/19
**Temp 1 – After 7 MORE days at 13ºC**	T1_7d_13C_Core	CHEESE	Core		8	12/9/19	12/9/19	12/9/19
**Temp 1 – After 28 MORE days at 13ºC**	T1_35d_13C_Rind	CHEESE	Rind	7	9	10/10/19	10/10/19	10/10/19
**Temp 1 – After 28 MORE days at 13ºC**	T1_35d_13C_Core	CHEESE	Core		10	10/10/19	10/10/19	10/10/19
**Temp 2 – After 7 MORE days at 20ºC**	T2_7d_20C_Rind	CHEESE	Rind	8	11	12/9/19	12/9/19	12/9/19
**Temp 2 – After 7 MORE days at 20ºC**	T2_7d_20C_Core	CHEESE	Core		12	12/9/19	12/9/19	12/9/19
**Temp 2 – After 28 MORE days at 20ºC**	T2_35d_20C_Rind	CHEESE	Rind	9	13	10/10/19	10/10/19	10/10/19
**Temp 2 – After 28 MORE days at 20ºC**	T2_35d_20C_Core	CHEESE	Core		14	10/10/19	10/10/19	10/10/19

Description of samples and timepoints used.

### 2.2. *k*-mer analyses reveal stable composition of cheese microbiome

A critical first step in the analysis of shotgun metagenome data is the co-assembly of sequencing data generated from each independent sample, into a resulting metagenome-assembled genome (MAG) catalogue, against which the taxonomic composition profiling of each sample can eventually be obtained. This process in turn requires a prior decision to be made about which data should be pooled for each co-assembly, as the computational efficiency of the process depends on balancing inclusion of too many samples with too diverse microbiomes, against inclusion of too few samples – both can give rise to a poorly assembled MAG catalogue. To inform this decision, we first analysed the raw FASTQ shotgun sequence data using KHILL, a novel *k*-mer counting method based on Hill numbers [14]. This approach enables computationally efficient exploration of sequence diversity directly from metagenomes, without requiring assembly or taxonomic profiling, even at shallow sequencing coverage. Fundamentally, KHILL is an information diversity-based technique that calculates the effective number of metagenomic samples given the distribution of *k*-mers between those samples. For pairwise comparisons, the metric therefore varies between 1 and 2. A KHILL of 1 indicates completely identical samples in terms of both *k*-mer identity and frequency. Completely unique, non-overlapping samples with no information in common would result in a KHILL value of 2 (two effective samples). Values in between (1–2) suggest partial overlap, as any *k*-mer overlap would lower the KHILL statistic. Initially designed to rapidly detect community shifts from a baseline, the approach has been previously used to efficiently detect temporal variation in SARS-CoV-2 viral strains sequenced from wastewater collected during the 2020 pandemic period [14]. This tool has two benefits in the context of our study, relating to the rapid insights it provides into the similarity/dissimilarity of the different samples. Firstly, it provides a rapid overview of the stages at which the community is changing (something that ultimately may represent an attractive tool for cheese producers who may wish to dynamically profile the microbiome community in their samples), and secondly these observations can be used to guide the co-assembly binning decisions.

**Fig 2:**
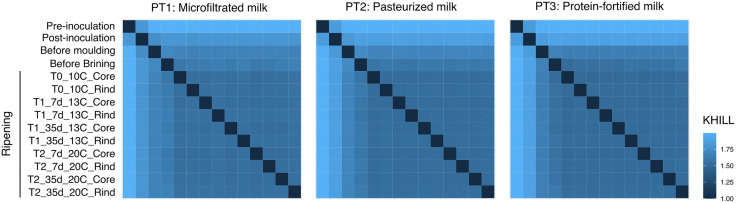
KHILL Heatmaps showing the similarity or differences in k-mer composition between the samples. The scale colour represents the intensity of the differences in KHILL observations. Darker intensities denote smaller differences in composition.

We therefore ran KHILL [14] for each of the 14 timepoints for the three PTs (Fig 1). We used 3 million reads randomly selected from each of the 14 sampling points in each PT as an input for KHILL and obtained correlation matrices shown in Fig 2. For all three PTs we see similar patterns, with the ripening phases after moulding (T0, T1, T2) being quite stable, with no major compositional changes.

In all PTs, principal differences observed were between the ripening phase (steps T0, T1, T2) and the first phases (“Pre-inoculation” and “Post-inoculation”; liquid milk sample), and “Before moulding” (curd, or grains) and “Before brining” (cheese sample). This can be attributed to fluctuations in microbial composition occurring at key processing stages. Specifically, we observe compositional shifts from raw milk (pre-inoculation) to pasteurised milk following the addition of starter cultures (post-inoculation). As fermentation progresses, further changes occur, culminating in the microbial profile observed in coagulated cheese grains (before moulding). KHILL comparisons between pre-ripening stages, and between these stages and ripening, reflect wholesale changes in the microbial composition in these early stages, and starter culture expansion in the cheese (KHILL ~ 2). Finally, we see the next shift just before entering the ripening process (Before brining). Note that all these changes happen in less than 10 hours, while the ripening process lasts for up to 35 days, which in this particular case informs us about the stability of the microbial composition along the ripening.

The above observations allowed us to form two conclusions. Firstly, the stability and low compositional changes at the ripening steps for all three PTs prompted us to generate a metagenomic co-assembly independently for each of the production trials in order to validate that, indeed, the composition is stable. Secondly, given that an estimated ~10–20% of milk and dairy production worldwide is lost due to bacterial spoilage [15] and thus there is an obvious interest in both closely controlling the production process, and being able to rapidly detect microbial changes that may affect the sample quality [16], KHILL may represent an interesting tool for integration into the cheese-making process. Specifically, as a result of the decreasing cost and increasing outputs of novel high-throughput sequencing technologies such as those marketed by Oxford Nanopore Technologies [17], they are beginning to be applied for real-time monitoring of environmental and industrial bioprocesses [18,19]. Unfortunately, however, the data generated conventionally requires considerable subsequent computational processing such as mapping reads to a database of choice. This is a time-costly bottleneck step that could be resolved by the application of the less computationally demanding KHILL method.

### 2.3. MAG diversity quantitatively confirms stability across cheese production trials

We generated 3 metagenomic co-assemblies from the data using the Anvi’o workflow for metagenomics [20,21], one for each of the three PTs, involving all 14 samples per PT (see Materials and Methods section, S1 Table in S1 File). Using the Anvi’o interactive interface we manually binned the data into 5 (PT1), 9 (PT2) and 10 (PT3) metagenomic bins, with a minimum of 36% observed completion, as estimated by the Anvi’o collection of single-copy core genes (SCG) (see Materials and Methods section) (Table 2). After the co-assembly, we obtained 411, 3,025 and 3,391 contigs respectively for the three PTs, with a length equal to, or longer than, 1 kb (Table 2). We retrieved a total of 18 different bins belonging to the *Bacteria* taxonomic domain (S2 Table in S1 File) for the three PTs, plus 6 bacteriophage-containing bins (discussed in section 2.4). Taxonomy up to the level of species was assigned by Anvi’o programme *anvi-run-scg-taxonomy,* using Anvi’o SCG collections [22], while the interactive manual binning was in process. Additionally, contig-level taxonomic classification was determined by Kaiju [23], and incorporated into the Anvi’o interactive visualisation.

**Table 2:** Co-assembly statistics.

ID	Total contigs	Longest contig	Estimated number of genes	Total contig length	N50	Anvi’o estimated bacterial genomes	Total selected bacterial bins	Total contigs assembled into bins	%
**PT1**	2952	137 kb	14207	11.97 Mb	10.18 kb	3	3	411	13.92
**PT2**	5163	136.80 kb	23228	20.01 Mb	7 kb	6	7	3025	58.59
**PT3**	5129	136.80 kb	24910	21.62 Mb	8.40 kb	7	8	3391	66.11

Basic statistics from the co-assembly process for each of the pilot production trials.

From these 18 bacterial bins we generated a final unique MAG catalogue, consisting of a total of 7 high-quality unique bacterial MAGs (Table 3). For that, we manually dereplicated the 18 bins from the three PTs (S2 Table in S1 File), by keeping those bins with >70% genome completion and <5% redundancy. In case of a tie, the longest genome was kept. Of note, amongst all the MAGs included in the final catalogue, only the *Janthinobacterium spp.* MAG from PT2, was under 95% in completion (completion = 71%). For both *Lactococcus* subspecies MAGs, their corresponding starter culture representative genomes were selected as representative MAGs, after phylogenomic analyses identified the strain from the cultures present in our cheese (see S1 Text, S5 Fig in in S1 Text).

**Table 3:** Statistics and taxonomy for co-assembled MAGs.

MAG name	Origin	Length (bp)	Contigs	N50	GC content	Completion (%)	Redundancy (%)	Species name
**CRE6**	*CULTURES*	*2510276*	*210*	*NA*	*NA*	*100.00*	*0.00*	*Lactococcus lactis cremoris*
**LAC2**	*CULTURES*	*2421935*	*152*	*NA*	*NA*	*98.59*	*0.00*	*Lactococcus lactis lactis*
**Leuconostoc_pseudomesenteroides**	*PT2*	*2001676*	*139*	*33436*	*37.45*	*100.00*	*1.41*	*Leuconostoc pseudomesenteroides*
**Clostridium_tyrobutyricum**	*PT3*	*2630280*	*627*	*5530*	*30.49*	*97.18*	*2.82*	*Clostridium tyrobutyricum*
**Janthinobacterium_spp**	*PT2*	*2567761*	*744*	*4550*	*62.19*	*71.83*	*2.82*	*Janthinobacterium spp.*
**Lacticaseibacillus_paracasei**	*PT3*	*2869405*	*357*	*15310*	*44.87*	*98.59*	*0.00*	*Lacticaseibacillus paracasei*
**Streptococcus_spp**	*PT3*	*1649953*	*200*	*15109*	*38.76*	*98.59*	*0.00*	*Streptococcus spp*
**Ceduovirus_1**	*PT2*	*22165*	*9*	*2851*	*35.26*	*100.00*	*NA*	*Lactococcus phage BIM BV-114, (predicted)*
**Ceduovirus_2**	*PT2*	*20592*	*6*	*4768*	*35.20*	*93.93*	*NA*	*Lactococcus phage bIL67, (predicted)*

Dereplicated MAGs and statistics.

We obtained the lowest number of bacterial MAGs (n = 3) from the microfiltered milk trial (PT1), with no taxa found to be exclusively present in this trial (Table 2, S3 Table in S1 File). This low number matches the expectation of the effect of the microfiltration process, by removing any potential opportunistic taxa in milk. Specifically, these 3 MAGs match the 3 expected bacteria from the added starter cultures, namely: the two subspecies of *Lactococcus lactis*: subsp. *cremoris* and *lactis* (see S1 Text), plus one strain of *Leuconostoc pseudomesenteroides*, although its abundance is < 1% out of all the reads recruited by the MAG catalogue.

In trials PT2 and PT3, both derived from milk that had not undergone microfiltration, we successfully assembled 7 and 8 MAGs, respectively. Of these, 7 were shared between both trials, including the 3 expected starter culture bacteria mentioned earlier. Following the MAG dereplication step, 4 unique MAGs detected in both PT2 and PT3 were retained in the final catalogue, along with the three from starter cultures, resulting in a total of 7 final bacterial MAGs. The 4 new MAGs identified in trials PT2 and PT3 were: *Lacticaseibacillus paracasei, Janthinobacterium* spp., *Clostridium tyrobutyricum* and *Streptococcus* spp. We were not able to identify *Streptococcus* spp. *and Janthinobacterium* spp. up to the species level. Genus *Janthinobacterium* is known to cause milk spoilage [24], while *Clostridium tyrobutyricum* (in the spore form) [25], confers a particularly undesired rancid smell and taste to cheese and can create late blowing [15]. *Clostridium* is also known as one of the causative agents for late blowing defects observed in semi-hard and hard cheeses [26] which can render the cheese not suitable for commercialisation. *Lacticaseibacillus paracasei* is considered a safe microorganism, naturally occurring through dairy processing [27]. Some members of the genus *Streptococcus*, like *Streptococcus thermophilus,* can be part of starter cultures, and are considered non-pathogenic [28,29]. While our results indicate that these bacteria are found at relatively low levels (<1%) throughout the process, we observed that their abundances consistently increase only after inoculation in pasteurised milk across PTs and remain stable during ripening (S1 Fig in S1 Text), discarding an initial presence in the milk. Despite their relatively low abundance, gene-expression and metabolic analyses would be needed in order to understand their true contribution towards the final properties of cheese. One additional bin, classified as *Pseudomonas* spp., was recovered from both PT2 (completion 36.6%) and PT3 (completion 60.6%). Although below the completeness threshold for high-quality MAGs, we report it given its relevance, as it confirms the low abundance of this potentially detrimental microorganism [30]. Most importantly, it remained undetected in the microfiltered milk trial PT1, which again demonstrates the effectiveness of milk microfiltration (S1 Fig in S1 Text).

### 2.4. Viral genomes

In addition to the 7 bacterial MAGs, we were able to recover from each of the three PTs, 2 further bins of non-bacterial origin, with their contigs classified by Kaiju [23] as *Lactococcus* bacteriophage viruses (Family *Siphoviridae*). Their average genome length was 19.4 kb (min: 12.9 kb; max: 23.2 kb) (S3-S5 Tables in S1 File), congruent with the size of a complete phage genome [31]. Initially, before dereplication and selecting them as MAGs, we named the bins *Ceduovirus_1* and *Ceduovirus_2*, as Kaiju classified one of the bins with this name, while the other bin was assigned to the category of *Unknown_Siphoviridae.* The *Ceduovirus* genus (alternatively known as “*c2*” virus) is one of the three most common groups of lactococcal phages, with a genome size of ~22 kb [32], which fits our results (S5 Table in S1 File), with the other 2 most common groups (named 936 [or *Skunavirus*] and P335 phages, respectively), both having larger genomes than *Ceduovirus* [31].

To estimate the completeness of the phage genomes we used CheckV, v.0.9 (db version 1.2) [33]. CheckV returned that completion for the bins was > 93% for 4 out of the 6 phage genomes detected (2 per PT trial), with the remaining 2 phages being assigned as 58% and 73% complete (S4 Table in S1 File). As we do not know the full taxonomic classification for the phages, and as both of them appear closely related, we dereplicated the genomes using the Anvi’o command *anvi-dereplicate-genomes,* which relies on computing average nucleotide identity (ANI) between all 6 phage genomic bins detected. Anvi’o determined that with at least 90%, and no more than 95% ANI, there were two different clusters. Anvi’o assigned exactly one bin from each PT to each phage group, fitting our hypothesis of having two distinct phage strains. We selected the two most complete genomes as representatives of each phage group, both obtained from PT2. These genomes had completion rates of 100% (22.2 kb) and 94% (20.6 kb), respectively, and were classified as “high-quality” by CheckV. They were subsequently added to our MAG catalogue, bringing the total to 9 unique species (Table 3). In an attempt to further determine the exact taxonomic identification of the phages, we ran BLAST v.2.2.5 [34] with our viral MAGs against the NCBI nt nucleotide database (accessed 22/08/2022), and we ranked results by the identity (>92%), %query coverage and length of the match (S1–S2 Tables). Top hits revealed two lactococcal phages, namely phage *BIM BV-114* and phage *bIL67* (Table 3).

### 2.5. A relatively low number of MAGs

Prior studies of the cheesemaking process based on metabarcoding analyses of 16S diversity report similar magnitudes of OTUs as those reported here. In one recent 16S based study, a maximum of 10 identified species were recovered from the surfaces of Époisses cheeses, both across ripening (28 days) and storage (90 days) [6]. A second study that profiled the microbiome of industrial cheese using 16S metabarcoding identified a median of 6.5 amplicon sequence variants per cheese sample [2], thus fitting our observations. In a third study that examined the microbial communities of washed-rind cheese produced from raw and pasteurised milk in an artisanal, non-industrially controlled process, researchers identified between 11 and 37 microbial species from the cheese rind [12]. Our results fall within the lower range of this analysis, which may be attributed to the shorter ripening times in our study. We did not detect a significant level of fungi in our reads (<0.01% of all the reads in PT3, as determined by Kaiju [23]. Several studies indicate that bacteria may outnumber fungi through cheese production [6,35], especially for Dutch style, semi-hard cheeses made with pasteurised milk, like the cheese we are studying, as their microbiome is usually not very complex [36].

### 2.6. *Lactococcus* spp. strains stably dominate throughout the ripening process

**Fig 3:**
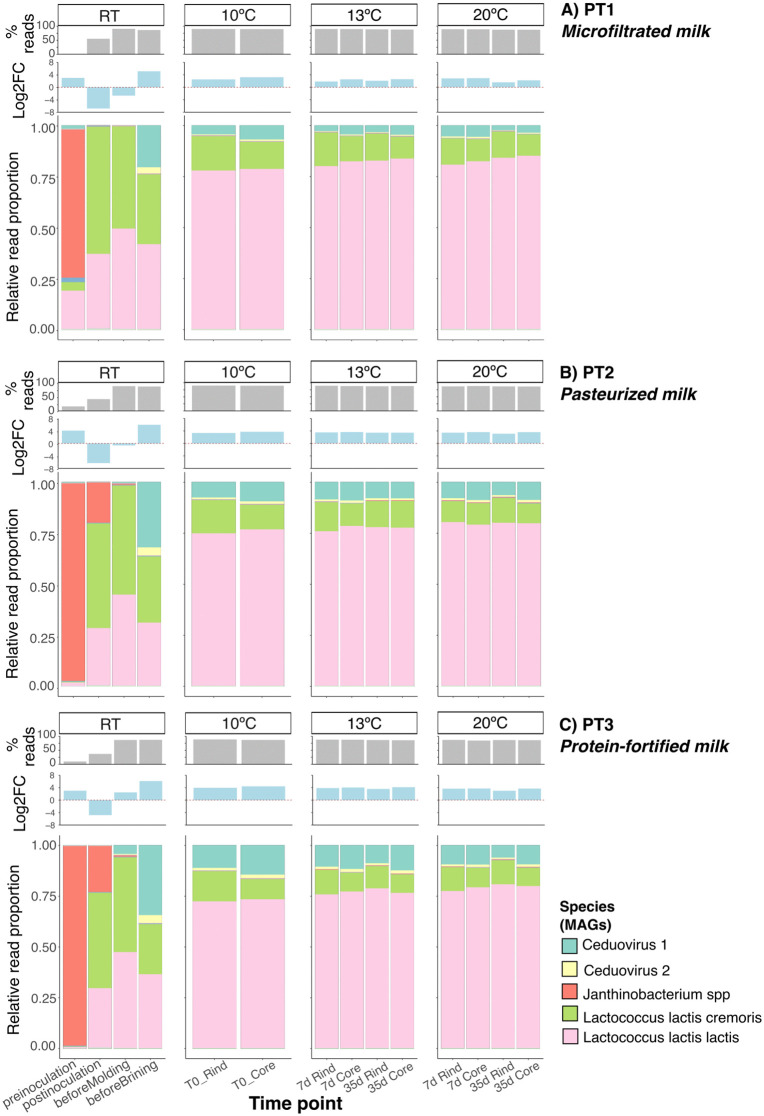
Relative abundance estimated as % of reads mapping to the 9 MAGs generated, for each of the three production trials (PT). **A)** Production trial for microfiltered milk, **B)** Production trial for pasteurised milk; **C)** Production trial for protein-fortified milk. In each of the A-C figures there are 3 panels. **Top panel:** percentage of reads from the raw data recruited by the MAGs. The “preinoculation” step recruits a low percentage of reads because of the initial low amount of microbe raw data at that step after host removal, as described earlier. **Middle panel:** log2 ratio for the proportion of phages to bacteria, estimated through coverage ratios with log-fold change applied. **Bottom panel:** Relative abundance for the 9 MAGs, in percentage of reads recruited.

After assembling a unique MAG catalogue, we sought to quantify the abundance of each of the 9 microbial species across the 14 key stages of the cheese production process. To do this, we mapped back our host-depleted raw metagenomic reads against the MAG catalogue, and quantified the relative abundance as percentage of reads recruited by each MAG. Broadly, for all PTs, we observed a relatively stable and constant trajectory for the abundances of MAGs, with principal domination by *Lactococcus*, as previously reported [2] (Fig 3). This aligns well with our previous analysis using *k*-mers (section 2.2), where we observed a high compositional stability through ripening time (Fig 2). As expected, at the pre-inoculation step only a small fraction of all the reads were recruited to the MAG catalogue (PT1: 0.6%, PT2: 16%, PT3: 9.9%) (Fig 3A-C). PT1 has a remarkably lower amount % of reads mapping back to the MAGs at this step, as this production trial was additionally microfiltered, theoretically removing any other milk microorganisms, despite the raw number of reads before being of comparable magnitudes: PT1: 244k, PT2: 306k, PT3: 177k (*in read pairs*). Immediately after the inoculation step, the percentage of reads mapped increased to ca. 40–50% for all three trials, with *Lactococcus* strains broadly dominating all the trials (Fig 3A-C). At this point, for all three trials, the proportion of *cremoris* over *lactis* subspecies had a -log2FC of ~ 1 (S2 Fig in S1 Text), meaning that the proportions of both are equally balanced. The trend is then inverted after the salt brining bath and *lactis* starts outnumbering *cremoris*, until stabilising at between 2-3x -log2FC, specially through the ripening process. The metabolic capabilities of ssp. *lactis* (S1 Text) may help to explain this differential thriving, as has been also previously reported [37]. The overall domination of lactococcal bacteria has also been recently reported in [6], with more than 70% of relative abundance for two *Lactococcus* subspecies *(L.lactis.* subsp. *lactis* and *L. lactis* subsp. *chungangensis)* through the ripening stages (28 days ripening). Incidentally, subsp. *lactis* seems also to be here the dominant one across the whole process.

Finally, for almost all of our time points beyond the “Before moulding” step, 85 to >90% (Fig 3A-C, top panel) of the reads are recruited to our MAG catalogue, which illustrates the fact that our MAG collection captures quite well the microbial communities in our pilot plant cheese samples.

### 2.7. Bacteriophage viruses are ubiquitous through the cheesemaking process

Throughout the whole process, we detected a considerable abundance of the 2 lactococcal phage MAGs (Fig 3A-C). Phages are known to disrupt starter cultures, particularly *Lactococcus* phages [38]. Notably, at least one phage strain was already present in the milk, with mean coverage values below 1X across all PTs, including microfiltered milk. This suggests that viable phages can persist despite both pasteurisation and microfiltration, albeit in reduced quantities [39]. To obtain an estimate of the proportion of bacterial cells to phage virus, we computed the relation between their genome coverages and turned it into a log2 fold change (log2FC). Immediately after the addition of starter cultures, most of the sequences generated consisted of *Lactococcus*, and the ratio of phage units to *Lactococcus* cells became negative, for all three PT (more bacteria than virus). It is not until the brining bath step that this trend is inverted, reaching a log2FC > 4, for all three PTs (Fig 3A-C, middle panel). Brining salt baths (see Materials and Methods section) are known to reduce the microbial complexity of cheese [40], thus this may have helped to slightly reduce the amount of phages, before entering the ripening stage. Then, once in the ripening process we observed a remarkably continuous and consistent ratio between phages and *Lactococcus* within trials. After the brining bath, and once cheeses enter the ripening process, the microfiltered milk PT1 showed a consistently lower proportion of phages to *Lactococcus* cells, with an average log2FC of 2.44, while for PT2 and PT3 it was 3.50 and 3.83, respectively (Fig 3). Unfortunately, we do not have replicates for the microfiltered milk PT, and thus we have no statistical power to demonstrate that microfiltration consistently reduces phage content, but we are confident this observation is worth reporting, as this relationship phage:bacteria was stable alongside the whole ripening process. We can conclude that microfiltration is not able to remove all phages in the milk, but our limits in experimental design do not allow us to prove whether they come from inside the factory facilities.

Both phage strains seem to have one particular *Lactococcus* strain as their target host. The proportion of abundances between the 2 bacterial hosts and the 2 viruses seem to correlate quite well in general terms and the correlation coefficient between proportions of both lactococcal strains and their respective phages are ~ 90%, while also significant (p-value < 0.05) (S3 Fig in S1 Text). We interpret this as that there is not an imbalance regarding the growth of phages or bacteria, which keep their respective growths linked, being remarkably significant during the ripening phase.

### 2.8. Cheese rinds and cores showed no lactococcal community differentiation

We quantified how different the rind and core communities of the cheese were in terms of *Lactococcus* communities. In the Herrgårds cheese system examined here, *Lactococcus* is the main bacterial group involved in the cheese final characteristics, so the sum of both *Lactococcus* strains’ abundances was used as a response variable in a two-way ANOVA analysis. ANOVA was run to determine whether the cheese sampling point (rind or core) has any effect on the abundance of *Lactococcus* recovered, and if this is different across trials. Through the ripening process (steps 5–9 in Fig 1) a total of 15 data points were collected per each sampling site (5 per trial) and the average *Lactococcus* abundances per group were calculated (S9 Table).

ANOVA assumptions were satisfied, as data did not deviate from normal distribution (Shapiro-Wilks test p-value = 0.99) and variances between groups were not significantly different (Levene test p-value = 0.27). An initial visual inspection of the data revealed that, in general, the rind tends to be richer in *Lactococcus* than the core (S4 Fig in S1 Text). ANOVA further demonstrated that there is both a significant effect (p-value = 3.27e-09), when looking at the PT influence alone (*i.e.,*: the type of milk) in the amount of *Lactococcus*, but also the abundance is different between the sampling sites (p-value = 2.93e-03). Interaction of *site:trial* was not significant (p-value = 0.59), meaning that the change in abundance is not particularly significant after the combined effect of both factors. Our analysis had a statistical power ~ 100% to detect an effect size as the observed for the PT factor at p-value ≤ 0.05 (Effect size: Ω^2^ for PT factor = 0.71; site factor = 0.07), but unfortunately our design does not have enough statistical power (~10%) to detect such a small effect size from the cheese sampling sites, despite being significant. Despite not being powered enough the abundance of *Lactococcus* was systematically higher for rind samples. Frequently, we find the opposite pattern in the literature, where lactococci species are less abundant in the rind than in the core [41]. In general, rind shows larger abundances of multiple microorganisms [42], being lactococci minoritarian, however the controlled conditions in our facilities may preclude the growth of alternative microorganisms, while allowing initial lactococci to thrive, explaining our systematic observation. Also, another possible explanation is that the ripening period for the cheese studied here is very short, leaving less time for other environmental microorganisms to colonise the rind.

### 2.9. Less abundant bacteria may also leave a big imprint

**Fig 4:**
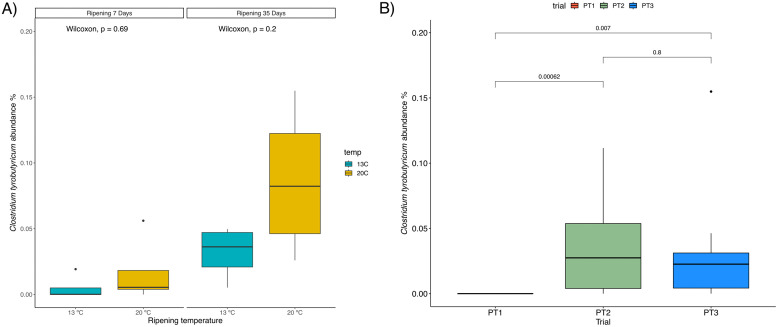
Abundance of *Clostridium tyrobutyricum.* **A)** Total % abundance of *C. tyrobutyricum* at each trial. Numbers in the plot connecting groups denote p-values derived from pairwise Wilcoxon-tests between trials. Y-axis represents % abundance of *C. tyrobutyricum* across the different PTs. **B)** The effect of ripening days and temperature on *C. tyrobutyricum* growth (% abundance). Numbers in the plot denote p-values derived from pairwise Wilcoxon-tests within ripening days categories.

Besides the landscape being dominated by *Lactococcus* strains, the remaining bacteria from our MAG catalogue are also present during the process. We recalculated relative abundances excluding both *Lactococcus* and viral MAGs (which dominate the dataset) and plotted percentages to better visualise the contribution of these 5 other minorly present bacteria, which have been also assembled into MAGs. On average, these minor MAGs represent less than 1% of the total composition of MAGs during ripening (S1 Fig, S10 Table in S1 Text). Right after the culture mix addition, *Leuconostoc pseudomesenteroides* becomes increasingly abundant and maintains a fairly constant abundance in all PT, even after 35 days of ripening. *Clostridium tyrobutyricum* is a bacterium that can be present in raw milk [43]. It shows up during later ripening stages conferring particular smell and taste to the cheese, which may not always be suitable for consumption and commercial production. We compared the abundance of *C. tyrobutyricum* at our PTs. First, we saw a highly significant reduction of *C. tyrobutyricum* in microfiltration trial (PT1) compared to PT2 and PT3 (average abundance ratio: 4768x more abundant in PT2 and 4721x in PT3 than PT1. Both comparisons were significant: Two-tailed Wilcoxon test p-value: PT1-PT2 = 6.2e-4 | PT1-PT3 = 7e-3) (Fig 4).

These results indicate that microfiltration removes nearly all *C. tyrobutyricum* spores present in the milk, which only starts developing in PT2 and PT3 after 21 days of ripening (S1 Fig in S1 Text).

The following analyses do not consider PT1, as we can confidently say that the amount of *C. tyrobutyricum* cells is nearly absent. We were interested in understanding *C. tyrobutyricum* growth patterns in relation to temperature and ripening days. Despite only having 4 data points at each temperature per number of ripening days, we found a clear trend (Fig 4B) showing that the high temperature set (20 ºC) promotes development of *C. tyrobutyricum*. This is confirmed by the fact that longer ripening times (35 days) show higher *C. tyrobutyricum* abundance than shorter ripening times (7 days ripening). At each group (temperature/days) shown in Fig 4B we have only 4 data points, so we lack statistical power to perform proper comparison within ripening days, despite the trend towards higher temperatures being remarkable.

However, after grouping *C. tyrobutyricum* abundances by ripening day, independent of the temperature factor, ANOVA tests showed significant differences (p-value = 0.028), while temperature as a factor is not significant (p-value = 0.13). The proportion of *C. tyrobutyricum* mapped reads represents a very minimal percentage of the reads per each PT during ripening (PT1: 0.0006%; PT2: 0.281%; PT3 0.28%, S10 Table in S1 Text). *Clostridium* vegetative cells do not survive pasteurisation, but we detected DNA derived from heat-resistant spores, and if the right conditions are given (time, temperature, etc.), they can develop into vegetative form causing large holes in the core of the cheese and undesired smell, as reported by our cheesemaking collaborators (L. T. Andersen, personal communication). As well, we cannot rule out the effect of other low-abundance bacteria, whether coming from our MAGs or not, which can be actively contributing metabolic volatile compounds to the final characteristics of cheese. In order to quantify that, transcriptomics and volatile compounds analysis would be necessary to gain a full understanding of a process which, *a priori* and compositionally speaking, seems quite stable.

## 3. Concluding remarks and perspectives

Our analysis of microbial composition during industrial cheese production reveals high stability and low diversity across the entire process, largely independent of milk pretreatment. This low diversity is primarily driven by the stable dominance of two distinct *Lactococcus* strains and their associated phages during ripening. Notably, microfiltration effectively eliminated undesirable bacteria (e.g., *Clostridium* and *Pseudomonas*), reinforcing its prophylactic role by ensuring that only the intentionally introduced culture microorganisms remain. Although present in low abundance, other bacterial species exhibited a consistent presence across all three production trials. To fully understand microbial dynamics—including both dominant and low-abundance bacteria—future studies should integrate metagenomics (DNA), metatranscriptomics (RNA), and metabolomics to achieve higher-resolution insights into how milk pretreatment affects the organoleptic properties of cheese. Our metagenomic analysis provides a valuable baseline framework, particularly for dairy producers interested in evaluating the effects of different milk bases. Unfortunately, we do not have replicates for the microfiltered milk PT and therefore lack the statistical power to conclusively demonstrate its benefits in consistently reducing low-abundance and undesirable species. Nevertheless, microfiltration also markedly reduces phage content, and we consider this observation particularly noteworthy, as the proportion phage:bacteria remained stable alongside the whole ripening process. We can conclude that microfiltration is not able to remove all phages in the milk, but our limits in experimental design do not allow us to prove whether they come from inside the factory infrastructures.

Additionally, our KHILL method, applied here to guide co-assembly, offers a practical application for the dairy industry. We clearly show that *k*-mer based approaches are a method to predict microbial composition of datasets. We were able to predict the stability of the three PTs during ripening. KHILL correctly predicted the turning point in the microbial composition, placing it before the ripening phase, but showing stability afterwards. Easily implementable in a factory setting, KHILL can rapidly assess batch quality and detect potential spoilage early in the production process, allowing for timely intervention.

## 4. Materials and methods

### 4.1 Sample collection

Sampling took place at the Arla Innovation Centre, in a pilot plant facility mimicking the industrial manufacturing process for Herrgårds cheese, located in Århus (Denmark), during 50 days between August and October 2019. We generated and compared microbiome profiles for three cheese production trials (PT; named here PT1, PT2, PT3), taking samples at the same processing time points for each of the three (n = 14 samples *per* trial; total sampling points = 42). The same raw source milk was used for all PTs, but treatment varied as follows: PT1: pasteurised + microfiltered, PT2: pasteurised only, and PT3: pasteurised + protein-fortified, in order to explore how these processes may shape the subsequent community. Pasteurisation was done at 72°C for 15 seconds. Microfiltration was performed using ceramic filters with a pore size of 1.2 µm (Tami Industries, Nyons Cedex, France). For the protein fortification process, milk was ultrafiltered to 4.2% target protein concentration, using a spiral membrane GR82 (Alfa Laval, Lund, Sweden). Protein fortification is a commercial procedure in order to reduce to a minimum the defects such as precipitation, flocculation and sandiness of milk, due to the high heat tolerance of proteins [44].

Sampling timepoints for each of the PTs, included (Table 1, Fig 1A; following numbers in parentheses are referred to numbers in Table 1): (1) The source milk prior to inoculation with the industrial starter culture that is a blend of two *Lactococcus lactis* strains: subsp. *cremoris* and subsp. *lactis*, and one *Leuconostoc pseudomesenteroides* strains. (2) Milk after culture inoculation, (3) curd before moulding and (4) cheese before salt brining. Then (5–9), from both the cheese core and rind were sampled at multiple time points during the ripening phase. The steps of the production were then standardised up to the ripening stage, where after an initial 14 days at 10º C (T0), each trial was subdivided into one ripening at 13º C (T1) and a second at 20º C (T2), in order to explore if temperature variation played a significant role on the microbiome community (Fig 1B).

For each PT, and once the cheese was pressed and brined, 5 samples were taken from the rind, and 5 samples were taken from the core, both using a sterile surgical scalpel. For the milk and grains samples in each PT (Table 1, total n = 4 samples) a volume of 3 ml was taken, and 200 µl were used to build the DNA libraries. Final cheese products were around 40 cm diameter and 12 cm of height. Whenever cheese samples were taken from the rind of the cheese, they were taken ~1 cm from the surface, while for the cheese core, they were taken at 2.5 cm deep from the surface, both on the same specific collection day for all three PTs.

Regarding the characteristics (% NaCl, time, temperature) of the brining bath for the cheese, Arla uses a proprietary specification not publicly disclosed. However, for reference, brining for most semi-hard cheeses is performed by immersion into brines composed of between 19–21% NaCl (w/w) at 10–14 °C for a few hours or several days, at a pH close to that of the cheese (5.2–5.3) [45]. In our PTs, all cheeses were exposed to the same brine conditions for the same duration and given that the aim of this study was not to compare different brining regimes but to assess microbial or compositional differences under standardised processing, we can confidently attribute observed variations to other experimental factors rather than to differences in salting conditions.

Each of the three PTs had a total duration of 49 days (from 20/08/2019 to 10/10/2019) from the time the milk was first delivered and processed. After the first ripening step (14 days at 10ºC), cheeses were split in 2 different lines, which were subject to two different ripening temperatures (13ºC and 20ºC) for a total of 35 additional days, taking samples at day 7 and day 35 of this specific ripening process. Samples had different consistency depending on the stage of the cheese process. The observed textures were liquid milk, coagulated cheese grains (curds) and solid cheese (Table 1). In order to obtain a liquid sample for further library processing from either the solid cheese or the coagulated cheese grains we liquified the samples using DNA/RNA Shield (Zymo Research, California, U.S.A.) stabilization solution.

### 4.2. DNA extraction and shotgun sequencing

DNA was extracted from 200 µl volumes of each cheese sample, using the ZymoBIOMICS MagBead DNA kit (Zymo Research) following the manufacturer’s instructions, with the exception of excluding the last washing step, and final elution into 60 µl of buffer EB (Qiagen, Hilden, Germany). Subsequently, the DNA was fragmented using a Covaris LE220-plus Focused-Ultrasonicator (Covaris Ltd., Brighton, U.K.), then converted into Illumina compatible shotgun sequencing libraries using the BEST protocol [46], prior to sequencing using 100 bp pair-end chemistry on an Illumina NovaSeq6000 platform (Illumina, California, U.S.A.) using Novogene’s commercial service (Novogene Europe, Cambridge, U.K.).

### 4.3. Bioinformatic analyses

#### 4.3.1. Sequence data preprocessing.

A total of ~2,200 million paired end (S1 Table in S1 File) reads were generated (PT1: 863M, PT2: 658M, PT3: 682M). Read trimming and adapter removal was done using fastp (v. 0.23.4) [47], using the default Illumina adapters for trimming. Host DNA (*Bos taurus*; domestic cow) was removed from the reads by mapping the raw reads with bowtie2 mapper (v. 2.5.0) [48] against the cow reference genome (ARS-UCD1.3, *bosTau9*), and selecting the unmapped fraction with samtools (v. 1.19) [49] (S1 Table in S1 File).

#### 4.3.2. KHILL *k*-mer analyses.

We used KHILL to do pairwise comparisons between each stage across all three samples. We randomly sampled 3 million reads from each stage and ran KHILL with default parameters. Since these comparisons are based on raw read libraries, we performed canonical *k*-mer counting without sketching, using all *k*-mer information contained in all 3 million reads.

#### 4.3.3. Metagenomics analyses.

We independently co-assembled each of the three sample production trials (n = 14 x 3 = 42 samples) using the Anvi’o snakemake workflow for metagenomics [20,21]. The workflow runs several software steps which allow the user to go from raw FASTQ to a contig database and a profile containing status from the contig mapping step needed downstream to complete other analyses with Anvi’o. The steps followed by the workflow base quality of the reads is checked by the software illumina-utils v. 2.12 [50]. MEGAHIT (v. 1.2.9) [51] was used with Anvi’o metagenomic default parameters and only considered assembled those having a length >1 kb. Mapper bowtie2 (v. 2.5.0) [48] was used to estimate coverage of contigs, by separately mapping back the FASTQs against the assembled set of contigs as reference sequences. At the end of the pipeline, a unique set of contigs (termed “contigs database”) and an Anvi’o profile with info like coverage and the tetranucleotide frequencies, was generated for each time point. The 42 profiles were merged, and together with the contig database constitute the starting point for downstream analyses and annotation with Anvi’o. Prodigal (v. 2.6.3) [52] was run once the contigs were assembled through Anvi’o to find genes by searching for ORFs, with Anvi’o default parameters. HMMER (v. 3.3.2) [53] was separately run through *anvi-run-hmms*, to identify and characterise genes from archaeal, protists, or bacterial origin, by comparing against single-copy core gene (SCGs) collections [22], using hidden Markov models (HMMs). These collections of SCGs were used to get completion and redundancy of MAGs or bins. Additionally, ribosomal RNA HMMs were included in the search through barrnap (https://github.com/tseemann/barrnap), as included in the Anvi’o HMMs collection. A custom HMM profile for RNAPol-A/B was used as input as an extra parameter to the Anvi’o *anvi-run-hmms* command to specifically annotate contigs for these two genes.

Given the low complexity of our samples, manual binning was done for each group of samples, after running *anvi-interactive*. The Anvi’o interactive interface displays a central unrooted phylogenetic tree for the contigs, and GC content, coverage and taxonomic assignment are displayed. We considered a bin to be a MAG when completion was > 70%, and redundancy was < 5%. In the case of a tie, the longest bin was retained.

#### 4.3.4. Taxonomy and functional analysis.

To determine the taxonomy of our bins, we relied on Anvi’o collection of SCGs, which is based on the Genome Taxonomy Database (GTDB) [54]. *anvi-run-scg-taxonomy* programme was run on the contigs database to annotate the SCGs present. Then, taxonomy assignment was done live during the manual binning process, by enabling the Anvi’o taxonomy option in the interactive interface and exported afterwards to table format through the *anvi-summary* option. Kaiju (v.1.9.2) [23] was run with NCBI’s non-redundant protein database ‘nr’ [55] as a reference to estimate taxonomy at the contig level. The Anvi’o tool *anvi-get-sequences-for-gene-calls* was first run on contigs to extract gene calls in FASTA format, which were input for Kaiju. Output was next integrated into Anvi’o interactive visualisation interface, as described elsewhere: http://merenlab.org/2016/06/18/importing-taxonomy/.

Krona (v. 2.8.1) [56] was used through function *kaiju2krona* from the Kaiju package over the Kaiju output, to convert the taxonomy into a hierarchical classification for the gene calls, for a manual overview of classified contigs. The interactive HTML Krona plots for manual exploration are provided as supplementary data (See S3 Dataset).

Classification of functions was done through Anvi’o (*anvi-run-ncbi-cogs*), by annotating the clusters of orthologous (COGs, version COG20) [57]. A script included in Anvi’o (*anvi-compute-functional-enrichment-in-pan*) was run to calculate the functional enrichment in the pangenome, for the 143 *Lactococcus* genomes. COG assignment to major functional categories was done through a custom R script, by parsing the resulting file from the command above for the COG20 “functions” categories. Enrichments between *L. cremoris* and *L. lactis* strains were calculated as log2 fold change; this is, the log2 for the quotient ratio between *L. lactis* and *L. cremoris* assigned COGs (S1 Text, S6 Fig in S1 Text).

#### 4.3.5. Viral classification.

Completion for viral MAGs was estimated by using CheckV (v. 0.9.0) [33], by running the *end_to_end* subroutine, using their native viral database v1.5.2. Dereplication for viral MAGs was done in two steps: i) we computed average nucleotide identity (ANI) for the viral genomes using pyANI [58], and ii) by using the Anvi’o function *anvi-dereplicate-genomes*, which returns the clusters and a representative MAG for each cluster. The standalone BLASTn algorithm as a command line interface [34] was run against a non-redundant NCBI database, and the best hits for our viral sequences based on sequence length and similarity were sorted and filtered.

#### 4.3.6. Relative abundances.

To calculate relative abundances of each of our MAGs, we mapped back the raw FASTQ reads to our unique MAG catalogue. We started by indexing the MAG catalogue in FASTA format with bowtie2 (2.3.5) [48] and then reads were mapped with MAGs as a reference file with default parameters. As a result of the mapping step, SAM format files were transformed into BAM compressed files by samtools (v. 1.9) [49]

Next, using Anvi’o, we generated a contigs database for our unique MAG catalogue, and a merged profile for all these samples that were mapped to MAGs. We now generated an Anvi’o summary for each trial (PT1, PT2, PT3), reporting in a table the coverage for each MAG in the catalogue, for each trial. Using custom R (v. 4.1) [59] scripts with the coverage as an input, we extrapolated the approximate number of reads that were recruited by each sample, and next we used a compositional normalisation (R package *microbiome*; [60]) to calculate the percentage of reads recruited by each MAG, which were plotted with R. The R package *phyloseqR* [61] was also used to handle relative abundances tables and facilitate plotting abundances.

#### 4.3.7. Statistical analyses.

Sample sizes for each of the PT were determined by selecting the 10 most critical steps during cheese production where microbial change would be likely to change. Statistical significance was determined at alpha level α = 0.05. All plots were generated using the *ggplot2* package [62] and the *tidyverse* group of functions [63] for R version v. 4.1. Basic two-way ANOVA analyses were performed with R (v. 4.1) [59]. Package *effectsize* [64] was used to estimate effect sizes and *pwr2* package was used to estimate statistical power for our design (https://cran.r-project.org/web/packages/pwr2/). No multiple testing corrections were applied, as our sample size precluded their meaningful application. All computer code used to process the data can be found at: https://github.com/pollicipes/Metacheese.

## Supporting information

S1 Text Phylogeny and metabolic analyses processes performed for the 2 identified *Lactococcus* MAGs. Includes supplementary figures S1-S6 and Table S10 as well referenced in the main text.
(DOCX)

S1 File S1-S6 Tables. Supplementary tables 1–6. Sequencing and assembly statistics for bacterial and viral MAGs included and used in this study.
(XLSX)

S1 Table Supplementary table 7. BLAST results for Ceduovirus_1 MAG.
(XLSX)

S2 Table Supplementary table 8. BLAST results for Ceduovirus_2 MAG.
(XLSX)

S3 Table Supplementary table 9. Read counts used to calculate relative abundances.
(XLSX)
