# Supplementary material for: The effect of different milk pretreatment methods on microbiome community development during Herrgårds cheese production and ripening
Source: PLoS One. 2026 Jul 14;21(7):e0350187. doi: 10.1371/journal.pone.0350187 (PMC13367665; doi:10.1371/journal.pone.0350187)
Supplement: Attachment — Submitted filename: Response_to_reviewers_Rodriguez_et_al_2026.docx [file pone.0350187.s006.docx]

**Point by point response to reviewers and comments, for the submitted manuscript:**

*“The effect of different milk pretreatment methods on microbiome community development during Herrgårds cheese production and ripening*”

(Rodriguez et al., 2026), for consideration at PLOS ONE.

See author answers in blue.

**Editorial comments to the author / Journal requirements** *(in italics)*

> We are very thankful to the editor for taking the time to revise and suggest improvements and guideline adherence for our manuscript. Please find below the point-by-point response to the requested tasks:

1. *Please ensure that your manuscript meets PLOS ONE's style requirements, including those for file naming.*

> We have now updated and corrected the format of the manuscript, according to the

formatting guidelines suggested. We re thankful for the thorough screening of the article format.

2. *Please state what role the funders took in the study. If the funders had no role, please state: "The funders had no role in study design, data collection and analysis, decision to publish, or preparation of the manuscript."* *Please include this amended Role of Funder statement in your cover letter; we will change the online submission form on your behalf.*

> We have now updated and corrected the role of funders in the study. As requested, we have

included an amended “*Role of Funders*” section in the Cover Letter accompanying the re-submission.

We are thankful to the editorial team for taking the time to doing this and subsequent changes described below in the online submission on our behalf.

3. *We note that you have provided funding information that is currently declared in your Funding Statement. However, funding information should not appear in the Acknowledgments section or other areas of your manuscript. We will only publish funding information present in the Funding Statement section of the online submission form. Please remove any funding-related text from the manuscript and let us know how you would like to update your Funding Statement.*

> As requested, we have removed the funding information from the manuscript and wrote the amended version in the cover letter, as requested.

4. *Thank you for stating the following in the Competing Interests section: “Lene Andersen reports a relationship with Novonesis that includes: employment. Christian Caroe reports a relationship with Novonesis that includes: employment. Søren Lillevang reports a relationship with Arla that includes: employment.”Please confirm that this does not alter your adherence to all PLOS ONE policies on sharing data and materials, by including the following statement: "This does not alter our adherence to PLOS ONE policies on sharing data and materials.” (as detailed online in our guide for authors http://journals.plos.org/plosone/s/competing-interests). If there are restrictions on sharing of data and/or materials, please state these. Please note that we cannot proceed with consideration of your article until this information has been declared. Please include your updated Competing Interests statement in your cover letter; we will change the online submission form on your behalf.*

> The “Competing Interests” section has been now updated and included in the cover letter.

**Comments to the Author** *(in italics)*

*Reviewer #1: Comments to authors:*

*My opinion is that it is a good study, which provides information about the microbial communities of a certain type of cheese made under controlled conditions, used very interesting tools in the study. Most importantly, it opens up the possibility of supplementingstudies in this field with other methods, other types of products and processing conditions, such as the production of cheeses made from raw milk.*

> We are thankful to this reviewer for taking the time to review the paper in such a detailed manner, and for appreciating the value that it contributes to PLOS One. The points raised and adressed will increase readability and clarity of our manuscript. Please find below in blue our responses to each of the points:

In particular:
Authors should review the format of the manuscript, especially the blank lines between paragraphs and paragraphs. In particular, review paragraph 2.9.

> Thanks for the thorough revision of these details. Paragraphs have now been fixed and distances/blank lines homogenized.

Line 87-91: The formulation of general and specific objectives would be expected along these lines, and a conclusion has been written. Please rewrite this content.

> A general objective and two specific ones have been added to the last paragraphs of the introduction, in addition to the conclusion already present (Lines 89-97 in the new updated manuscript).

In addition, I believe that the following phrase "even if production is moved to different locations or different milk bases and pretreatments are applied" is not rigorous; it cannot be assured that the results were completely repeatable in other industries. This is confirmed by those discussed in 477-484: the brining is specific to this factory, so it could have an impact on the results, especially of rind. This study should be completed in other cheese factories, to see that factor of variation.

> We are very thankful to the reviewer for pointing this out. Certainly, it is something that we cannot state, given the characteristics of the study. We have now toned down and removed this statement, which certainly does not add value here and may confuse the reader.

Line 364: Change “…samples, Frequently…” to “…samples. Frequently…”

> This has been done.

Line: 363-368: The authors should consider the possibility that the abundance of Lactococcus in core or rind in this study is different from other studies, because it is this study the period of maturation studied is very short.

> This is a very interesting suggestion that we did not realise at the time of writing, but which makes total sense, in line with the proposed explanations of more hygienic condition in an industrial pilot plant, to explain the high abundance of *Lactococcus.* This explanation has been added to the text in lines 388-390 of the revised version of the manuscript. We are thankful for this suggestion.

Line 394: Format error: align to the left.

> Solved. All figure legends have now been standardised, regarding paragraph layout justification.

Line 487: Are 49 or 42 days from the time the milk was first processed?. Throughout the text it is not clear what is the time of ripening; please review and clarify.

> We apologise for the confusion. Certainly, this is something that needed clarification. We have now updated and clearly stated in the methods the exact amount of days through which the experiment was run (49 days). We are thankful to the reviewer for pointing this out, as this is key to understand the process.

**Additional author’s comments:**

> In this revised version of our manuscript, we have now included the accession codes to download all the used raw FASTQ data from the European Nucleotide Archive (ENA) (publicly available), together with the required metadata. Additionally, a Zenodo citeable repository has been created to store the Additional Datasets, and

> Also, other very minor edits to the orthography/grammatic of the manuscript text (marked up with track changes), have been added to the manuscript (e.g. “*specialy*”; wrong spelling changed to correct form “especially”, typos were corrected, etc...)

We hope all these changes contribute to satisfy the reviewer and editorial requests, and improve the manuscript for publication.
